# The Relationship between Trabecular Bone Structure Modeling Methods and the Elastic Modulus as Calculated by FEM

**DOI:** 10.1100/2012/827196

**Published:** 2012-05-02

**Authors:** Tomasz Topoliński, Artur Cichański, Adam Mazurkiewicz, Krzysztof Nowicki

**Affiliations:** Faculty of Mechanical Engineering, University of Technology and Life Sciences, Kaliskiego 7 Street, 85-789 Bydgoszcz, Poland

## Abstract

Trabecular bone cores were collected from the femoral head at the time of surgery (hip arthroplasty). Investigated were 42 specimens, from patients with osteoporosis and coxarthrosis. The cores were scanned used computer microtomography (microCT) system at an isotropic spatial resolution of 36 microns. Image stacks were converted to finite element models via a bone voxel-to-element algorithm. The apparent modulus was calculated based on the assumptions that for the elastic properties, *E* = 10 MPa and *ν* = 0.3. The compressive deformation as calculated by finite elements (FE) analysis was 0.8%. The models were coarsened to effectively change the resolution or voxel size (from 72 microns to 288 microns or from 72 microns to 1080 microns). The aim of our study is to determine how an increase in the distance between scans changes the elastic properties as calculated by FE models. We tried to find a border value voxel size at which the module values were possible to calculate. As the voxel size increased, the mean voxel volume increased and the FEA-derived apparent modulus decreased. The slope of voxel size versus modulus relationship correlated with several architectural indices of trabecular bone.

## 1. Introduction

Numerous papers have evaluated the mechanical properties of bone and have presented the opinion that these values can be determined not only by bone density but also by the properties of single trabeculae and the structure of the trabecular part of the bone [1–3]. In clinical practice, one of the most commonly applied methods of evaluating bone density is dual energy X-ray absorptiometry (DEXA). This method allows the determination of bone mineral density (BMD) and bone mineral content (BMC) as demonstrated by Hansson et al. [4], McBroom et al. [5], Beck et al. [6], and Cody et al. [7], which are used to indirectly determine fracture risk. Investigating density with DEXA is favorable because it is quick and the results are available immediately. However, the disadvantage of this method is that it does not consider the complexity of the structure of trabeculae.

To evaluate the structure of trabecular bone in vitro, computer microtomography (microCT) is commonly applied. This makes it possible to generate bone images with an accuracy of 6–8 microns. Based on images of the inner part of the bone obtained as a result of such investigations, micromechanical models of the bone structure subject to finite element (FE) calculations can be developed [8–11]. However, the application of this technique in vivo in clinical practice is difficult. The disadvantage of this technique is that it is time consuming, depending on the assumed performance accuracy. Currently, it is impossible to apply microCT to patients.

In clinical practice, it is possible to image trabecular bone using techniques such as multidetector computer tomography (MDCT) or high-resolution magnetic resonance imaging (HRMRI). The resolution of images obtained using these techniques is about 100–300 microns at a slice density ranging from 300–500 microns [12–16]. At present, however, the availability of these techniques is limited.

Time limitations are one of the major problems of medical investigations because X-ray investigation is harmful and requires the patient to stay motionless. In such cases, limiting the time of the medical investigation is beneficial for the patient, but this usually leads to a reduction in the resolution of the test.

In numerous papers, the effects of increasing the voxel size of the FE model to a larger size than that obtained from microCT on the calculated values indices of the structure [17–20] and on the strength indices [9, 17, 21–25] of trabecular bone have been studied. Kim et al. [25] found that for most of the structure indices, the results from analysis of images with larger voxel sizes, such as 110 microns, were correlated with results for a 21 microns voxel size. Cendre et al. [26] found that the HRCT (high-resolution microtomography) system with 150 microns resolution is not sufficient to predict the true values of the structural parameters measured by histomorphometry. Tabor [27] presents a new method of analysis called “optimal path” analysis of grey-level magnetic resonance imaging (MRI) images for improved estimation of the Young's modulus of trabecular bone samples.

The aim of our study is to determine how an increase in the distance between scans changes the elastic properties as calculated by FE models. The basis for the construction of our models is a set of scans of trabecular bone. Sample models were obtained from a microCT investigation. In our study, we assumed a simulated resolution change by altering the dimension of the voxel size in the direction of the axis of the sample. This simulation was based on the omission of some scans from the original data set in accordance with the proposed methodology.

The main advantage approach to modeling proposed by the authors is that models can be created based on a smaller microCT data set in comparison with modeling methods proposed by other authors. Consequently, in clinical practice, data collection will take less time and therefore will be limited dose of X-ray radiation absorbed by the patient.

This work also examines the relationship between the calculated modulus and the values of indicators describing the structure of bone.

## 2. Material and Methods

**Figure 1:**
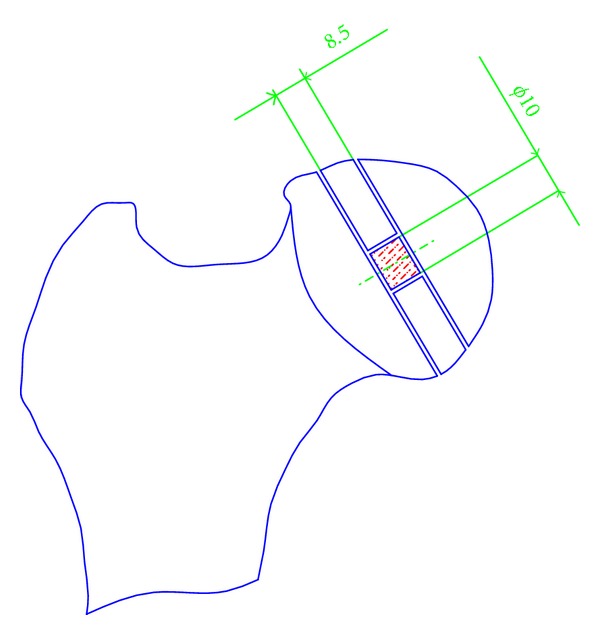
Trabecular bone sampling method. The sample axis coincided with the axis of the head and neck of the femur.

Cylindrical samples of 10 mm in diameter and 8.5 mm long were taken from the epiphyses of the heads of human femurs perpendicular to the axis of the neck, as shown in Figure 1 [28]. The study was approved by the Local Ethic Committee.

The heads were derived from the bones of patients after osteoporotic or coxarthrotic fractures and hip joint replacement surgery. The samples were investigated with a *μ*CT80 microtomograph (Scanco A.G., Switzerland) to obtain images of the inner part of the sample and to measure the selected indices of the structure: bone volume ratio (BV/TV), trabecular thickness (Tb.Th), and trabecular number (Tb.N) proposed by Parfitt et al. [29]. For each sample, an average of 230 microtomographic slices with a slice increment of 36 microns was obtained (parameters: 70 kV, 114 *μ*A, 500 projections/180°, 300 ms integration time). Measurements were made for 21 samples derived from bones diagnosed as osteoporotic (o) and for another 21 samples from bones diagnosed as coxarthrotic (c). For each sample, a basic geometric model was built as a comparative model.

Basic model development was based on a set of 230 binary images obtained using microCT that presented the structure of the sample in layers perpendicular to its axis. The size of the pixels in these images was 36 × 36 microns. The basic models were developed by creating voxels representing the bone, such that pixels on the same coordinates in both images had to be the color representing the bone.

The Hexahedral (H) method was used as a reference, as described in the literature by Ulrich et al. [30]. Three additional different methods of model simplification were proposed, First-Last (F-L), First-Second (F-S), and First-Third (F-T), all of which included limiting the number of scans. The main difference between these approaches was the method of using the scans for layer selection for voxel building.

### 2.1. The Hexahedral Method

In the first approach, fragments of the basic model in the shape of a cuberrille with a size equal to a multiple of 36 microns were analyzed for a range of side length of 72–288 microns.

**Figure 2:**
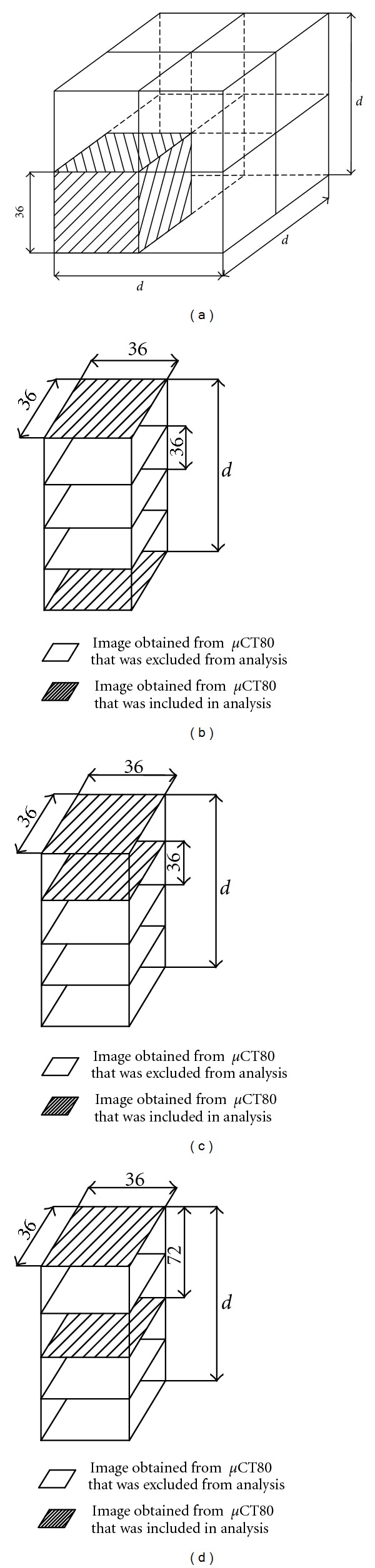
Voxel structure diagrams of length *d* using the (a) Hexahedral, (b) First-Last, (c) First-Second, and (d) First-Third methods.

Whenever more than 50% of the cuberrille volume was filled with voxels representing the bone (Figure 2(a)), a new voxel representing the bone was created with the dimensions of the cuberrille analyzed. In this approach, the basic model was built based on the original set of images obtained from microCT, and thus no data reduction (concerning the number of images) was needed to build the simplified model.

Simplification using the subsequent methods involved limiting the number of images used to build the model compared with the original number of images obtained with microCT.

### 2.2. The First-Last Method

To decrease the amount of data (the number of images) needed to build the model, the set of data obtained with microCT was limited. The difference, compared to the first approach, involved disregarding images of selected layers of the sample. In this model-simplification method, the voxel length (*d*) was changed along the sample axis. This simplification involved building the model layer with two images of layers a multiple of 36 microns apart for a range of voxel length of 72–1080 microns. The condition for creating a voxel representing the bone was the same as for the basic model. The voxel created was 36 microns ×36 microns ×*d*; the value *d* was included in the range described above. The voxel structure diagram for this approach is given in Figure 2(b).

### 2.3. The First-Second Method

In the third approach, two successive images of layers obtained with microCT 36 microns apart were compared, after which a voxel of a predefined size “*d*” was created. The value “*d*” varied within the range of 72–1080 microns with a step size of 36 microns. This voxel development method is given in Figure 2(c).

### 2.4. The First-Third Method

In this approach, two images 72 microns apart were used. The voxel created was 36 microns ×36 microns ×*d*, where the value of d varied within the range of 72–1080 microns with the step size of 72 microns. This voxel development method is given in Figure 2(d).

Numerical analyses were performed with the FE method using ANSYS Academic Research R12 (Ansys Inc., USA). The division network characteristic for the method used was prepared such that it constituted a direct transformation of the geometric notation of the structure considered in the “voxel-to-element” method [30]. The analyses used 3-D 8-node SOLID45 elements with a side length of 36 microns. A single regular voxel (the first approach) corresponded to a single 3-D finite element. If the geometric model was built from prolonged cubicoid voxels (as in F-L, F-S and F-T methods), each voxel was replaced with multiple 3-D finite elements. Due to the stability of the process of iteration of the calculations when solving the numerical task from the division network, elements poorly connected with rest of the model and not affecting the rigidity of the structure were eliminated.

For the purpose of the analyses, which were linear, homogenous material properties *E* = 10 GPa and *ν* = 0.3 were assumed [30]. Under such conditions, the results of the analyses reflected only the number and distribution of the respective cubes in the tissue-modeling structure.

In defining the boundary conditions in each approach, one base of the cylindrical sample was given zero displacement towards the axis. The opposite base was given a kinematic excitation acting along the axis of the cylinder to obtain the assumed compressive deformation *ε* = 0.8%. The result of the calculations was the compressive axial force (*F*) needed to obtain this assumed deformation. Based on these results, the elastic module (*E*) was calculated.

Statistical analyses were performed with software R (The R Foundation for Statistical Computing).

## 3. Results

Table 1 presents the range of values, mean value, standard deviation (SD), and relative standard deviation (RSD = mean  value/SD) of the selected indices of the sample structure obtained from microCT.

**Figure 3:**
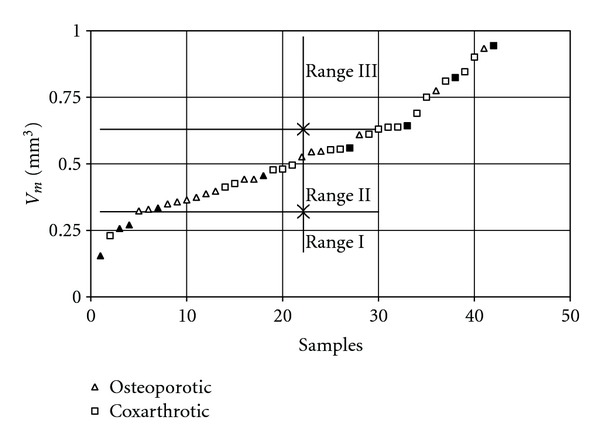
Increasing values of *V*_*m*_ for all samples.

The results indicate a wide range of variation and large values of standard deviations. The mean volume (*V*_*m*_) of the samples, representing the bone of respective layers of the basic models, is given in Figure 3 according to increasing values. The curve of the V_m_ demonstrates three ranges. The selection was carried out using an independent description of the fragment ranges as indicated by a straight line using a linear regression method. The first range is dominated by the samples cut from the bone diagnosed as osteoporotic, and the third range is dominated by those diagnosed as coxarthrotic. It was observed that there are samples associated with a beginning, middle, and end of each of ranges. Hence, further analyses involving 9 samples, of which 5 were derived from osteoporotic bone and 4 from coxarthrotic bone, were necessary. Selected samples from each range are noted in Figure 3. For these samples, the calculations were done as an example, and the results of samples from the middle of each range were shown graphically.

**Figure 4:**
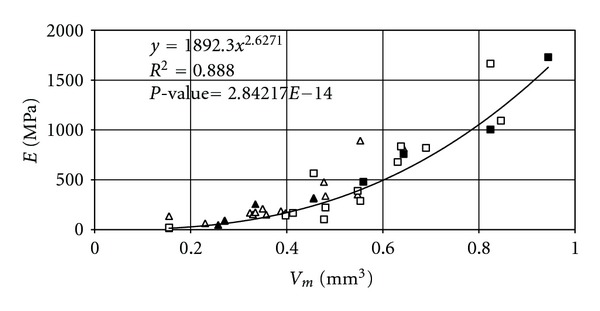
Dependence of the elastic modulus on the mean volume *V*_*m*_.

 For the basic models based on data from microCT, with voxels of 36 microns in size, the elastic modulus versus mean volume is presented in Figure 4. Interestingly, there is a strong relationship between these parameters, which confirms the possibility of performing full evaluations for selected samples. Figure 4 also shows selected samples marked according to Figure 3 for which the relationship between *E*  and  *V*_*m*_ has a high coefficient of determination (*R* ^2^ = 0.96).

**Figure 5:**
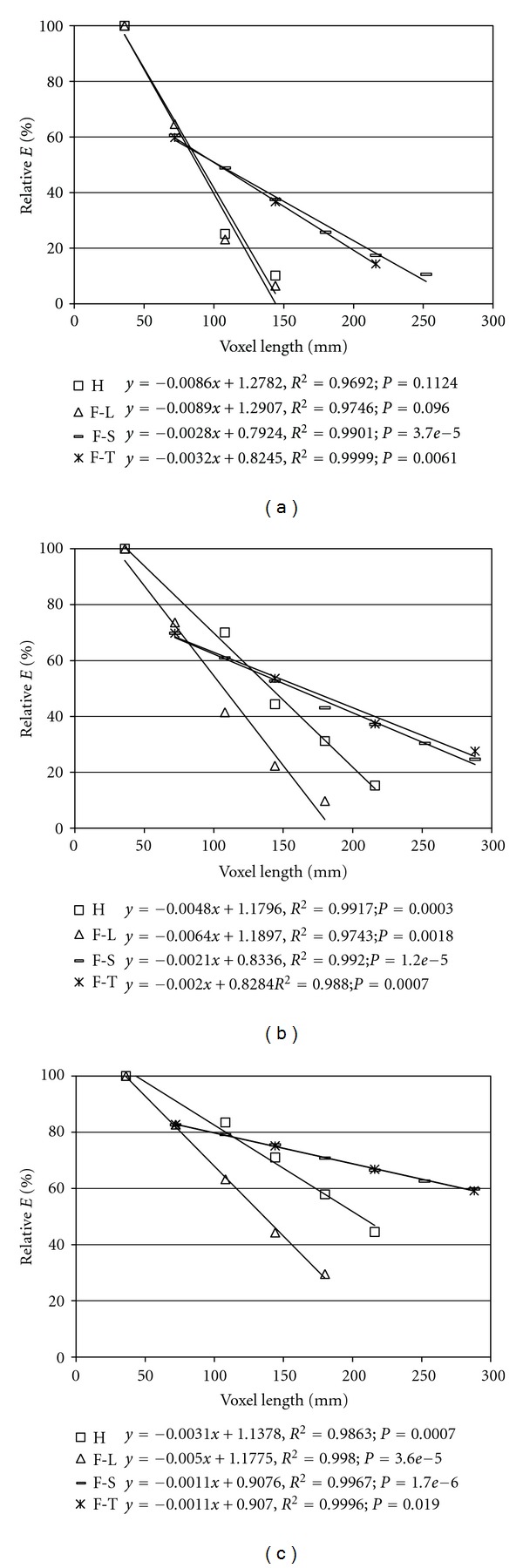
The results for the three samples simplified by four different methods (a) o34, (b) o38, and (c) c24.

The Relative modulus (Relative *E*) was calculated, which is the elastic modulus obtained from the calculations for a given model relative to that obtained for the basic model (voxel size 36 microns). Graphical calculations are presented for three representative samples from each of the ranges investigated. The representative sample from the range of lowest volume is marked as o34, that from the middle range as o38 and that from the greatest-volume range as c24.  Figure 5 presents the relative module calculated for models built for the same samples with successive simplification methods.

The coefficient of determinacy for the relative moduli determined for all of the samples and structural simplification methods considered (Figure 5) is high, ranging from 0.92–0.99, which confirms a strong relationship between the modulus and the voxel length assumed for FE analyses.

Regression lines determined using the H method (Figure 5), the F-L layers method and structural simplification methods give relatively high absolute values of regression coefficients, except the results for the F-L method for sample c24. The range of effectiveness of these methods is, in practice, determined with voxels having a length in the range of 100–150 microns. Regression lines determined with the F-S method and the F-T method of structure simplification show almost threefold lower absolute values of regression coefficients compared with the previous two methods, outside of the c24 sample as well.

Based on a statistical test of the hypothesis conducted for equality of intercept and regression coefficient equality for the F-S and F-T methods, the value of *α* = 0.005 cannot be denied.

It is interesting that for larger values of *V*_*m*_, that is, samples comprising higher levels of bone tissue, simplification of the structure has less influence on the calculated value of *E*. This is visible for the sample c24 for the F-S and F-T methods, where the value of Relative *E* versus voxel length reaches 60%.

The range of effectiveness of these methods is, in practice, determined with voxels having a length in the range of 200–250 microns.

**Figure 6:**
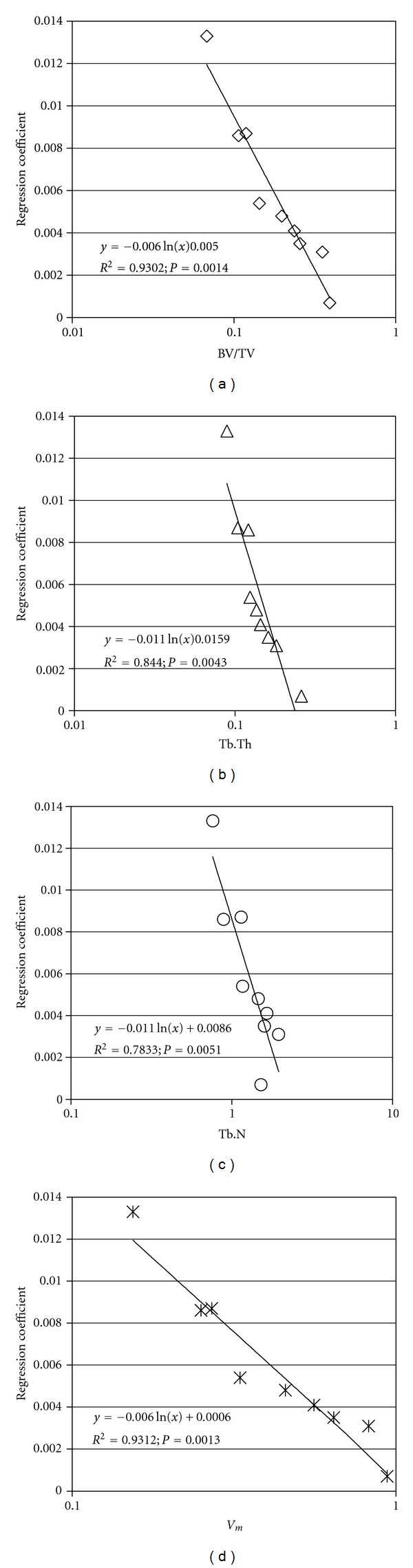
Relationships between the regression coefficients calculated with the hexahedral method of simplification and the structural indices (a) BV/TV, (b) Tb.Th, (c) Tb.N, and (d) *V*_*m*_.

Figure 6 presents the relationships between the regression coefficient calculated using one structural simplification method, the H method, and the selected structural indices. To plot these diagrams, the relative modulus for a voxel size up to 300 microns was calculated for each simplification method, and its logarithmic approximation was made. Then, the relationship between the absolute value of the regression coefficient and a given indices of the structure was established.

The relationships between the relative modules calculated using four structural simplification methods and the selected structural indices are given in Table 2. The diagrams, for all simplification methods, were created with the convention on Figure 6; the slope (*a*) and the intercept (*b*) of the regression lines were read. These values, together with the corresponding determination coefficients, *R* ^2^, are given in Table 2.

In the course of this analysis, we refer to the relationship between the regression coefficients (for the relative modulus) and the selected indices of the structure of the bone. For all of the structural simplification approaches analyzed, the relationship between BV/TV and *V*_*m*_ was found to have the highest degree of occurrence. For the H and the F-L methods, achieved determination coefficients were up to 0.93. For the F-S and the First-Third methods, determination coefficients of up to 0.74 were obtained.

## 4. Discussion

Our model of the bone structure involved the use of microtomographic scans 36 microns apart. This distance falls within the most frequently applied range, namely, from 6–8 to 60 microns [31], although recently, distances of 10 microns [32], 20 microns [33, 34], and 82 microns [35] have been commonly applied. The scanning resolution can significantly affect the structure modeled and thus influence the results of the calculations of mechanical properties [36].

In our work, we used 3-D 8-node hexagonal elements, as reported by Kosmopoulos et al. [37]; Bevill and Keaveny [34]; Mazurkiewicz and Topoliński [28]. In other studies, different types and dimensions of elements are used in analyses of the structure of bone. Four-node elements [38] and regular 2–D honeycomb [39] have been used in other research studies of trabecular bone. One report proposes a simplified approach to modeling the variation in trabeculae geometry with bar-and beam-type finite elements, with the analysis considering the variation in the direction, length and thickness of the trabeculae [40]. Similar considerations have been reported by other authors, who modeled each rod-like trabecula with one thickness-matched beam and each plate-like trabecula with several beams [41]. Calculation using 1-D or 2-D elements can lead to excessive simplification of the model and reduce exactness of the calculations.

In the analyses performed in this paper, it was assumed that the trabecular bone tissue element is isotropic and linearly elastic (*E* = 10 GPa, *ν* = 0.3), as reported by other authors [27, 37, 42]. We are aware that other approaches have been applied. For example, besides the standard approach, heterogeneous FE models have been proposed, for which the value of the tissue modulus was approximated from the degree of mineralization of bone (DMB) [43]. In the above heterogeneous FE model, calculations of the elastic modulus of human trabecular bone under axial compression are considered, which is a common load case in such calculations [28, 30, 31].

In this paper, four methods of changing the voxel size of the bone model structure are considered. In the first method, the size of the voxel was changed at unchanged criteria of bone identification, and in the succeeding three, the simplification was carried out by limiting the number of scans. The latter cases can lead to similar calculation results using less data than the modulus calculations without simplifications. To build all of the models, we used binary images obtained after thresholding original data obtained from *μ*CT80. A threshold value was determined based on the settings of the devices.

In general, all of the calculation methods proposed lead to decreased values of the calculated modulus. As expected, the greater the simplification, the greater was the decrease in the modulus. The simplification of the structure following the Hexahedral method results in smaller decreases in the modulus. The results of the calculations using this method are in agreement with results presented by Bevill and Keaveny [34]. These other results which corroborate our studies performed calculations of mechanical properties of trabecular bone for vertebral, femoral neck, and greater trochanteric specimens. Calculations included the elastic modulus and the yield stress, based on data from *μ*CT20 with 20 microns resolution. These quantities were calculated for voxel sizes of 40 microns, 80 microns, and 120 microns. In previous work [34], which corresponds to the first approach presented here, comparable results for the elastic modulus are reported for the range of 20–80 microns.

Smaller decreases in the elastic modulus are obtained with the First-Second and First-Third methods. These results do not differ from each other, demonstrating that the simplification method assumed in these approaches does not cause a loss of essential information on the structure of the bone modeled. Performing further research with that method, such as searching for the maximum voxel length not causing significant changes in the elastic modulus, will affect the potential of using this research in vivo.

In each case, the decrease in the modulus increases with a greater degree of structural damage. In this paper, the Vm is used, and thus the lower the Vm, the greater the decrease in the modulus. In the work [34] similar conclusions are reported for BV/TV. The decreases in the elastic modulus are a linear function of voxel size, and the coefficients of determinacy are above 0.96. An increase in voxel size is accompanied with a decreased module. This decrease is more significant for smaller values on BV/TV. Thus, inaccuracy in the calculation of the Young modulus is connected with the structure of the sample.

Application of the method of F-S and F-T allows for better representation of the strength of the bone compared to the H method. For F-L method, obtained results were less accurate than those compared to the H method.

The Hexahedral and First-Last methods are useful for a smaller voxel size in comparison to the First-Second and First-Third methods. In both cases, modulus correction should be done, depending on the structural bone parameters. If the bone sample is more porous, greater correction should be done.

For the Hexahedral and First-Last methods, there are strong relationships between the structure indices and the values of the regression coefficients (directional) obtained from the plots of *E* versus voxel length for voxel size of 200–250 microns. For the other methods, these relationships are satisfactory for the voxel length 100–150 microns.

In comparison to the First-Second method, the First-Third method gives a better relationship between module and structural indices of bone, despite the higher distance between scans.

The statements above apply to all samples from both groups of bones. While better results are obtained for samples comprising higher levels of bone tissue.

## Figures and Tables

**Table 1:** Values of structure indices obtained from microCT measurement.

Indices	Min-max	Mean value	SD	RSD, %
BV/TV, —	0.068–0.392	0.222	0.079	36
Tb.Th, mm	0.089–0.259	0.151	0.036	24
Tb.N, mm	0.76–1.956	1.436	0.267	19

**Table 2:** The relationship between the coefficients of determination (from the graphs RE versus voxel length) and structural indices of bone.

Method	Indicator	*a*	*b*	*R* ^2^
H	BV/TV	−0.006	−0.005	0.9302
	Tb.Th	−0.011	−0.0159	0.8440
	Tb.N	−0.011	0.0086	0.7833
	*V*_*m*_	−0.006	0.0006	0.9312

F-L	BV/TV	−0.003	0.0004	0.8071
	Tb.Th	−0.006	−0.006	0.8126
	Tb.N	−0.005	0.0076	0.6017
	*V*_*m*_	−0.003	0.0034	0.8100

F-S	BV/TV	−0.00008	0.0003	0.4414
	Tb.Th	−0.002	−0.0014	0.4981
	Tb.N	−0.001	0.0019	0.2808
	*V*_*m*_	-0.00007	0.001	0.4321

F-T	BV/TV	−0.002	−0.0019	0.7469
	Tb.Th	−0.004	−0.0057	0.6162
	Tb.N	−0.004	0.0032	0.6935
	*V*_*m*_	−0.002	0.0002	0.7406
